# Pulsed Field Ablation: A Comprehensive Update

**DOI:** 10.3390/jcm13175191

**Published:** 2024-09-01

**Authors:** Fatima M. Ezzeddine, Samuel J. Asirvatham, Duy T. Nguyen

**Affiliations:** 1 Department of Cardiovascular Medicine, Mayo Clinic, 200 First Street SW, Rochester, MN 55905, USA; 2 Department of Pediatric and Adolescent Medicine, Mayo Clinic College of Medicine, Rochester, MN 55905, USA; 3 Department of Biomedical Engineering, Mayo Clinic, Rochester, MN 55905, USA; 4 Department of Clinical Anatomy, Mayo Clinic, Rochester, MN 55905, USA

**Keywords:** arrhythmias, catheter ablation, electroporation, pulsed electric field, pulsed field ablation

## Abstract

One of the recent advancements in the field of cardiac electrophysiology is pulsed field ablation (PFA). PFA is a novel energy modality that does not rely on thermal processes to achieve ablation which, in turn, results in limited collateral damage to surrounding structures. In this review, we discuss the mechanisms, safety, efficacy, and clinical applications of PFA for the management of atrial and ventricular arrhythmias. We also summarize the published pre-clinical and clinical studies regarding this new technology.

## 1. Introduction

Electroporation involves the application of strong pulses of electric fields over a short period of time (micro- or nano-seconds). This results in disruption of cellular homeostasis, increase in cell membrane permeability, apoptosis, and cell death. The earliest clinical applications of electroporation included electrochemotherapy, gene transfer, and the ablation of solid tumors. In recent years, electroporation has earned substantial attention in the field of cardiac electrophysiology and has been shown to be a promising technique for ablation of cardiac arrhythmias. In this review, we discuss the mechanisms of pulsed field ablation (PFA) and review the evidence behind its safety and efficacy for the management of atrial and ventricular arrhythmias.

## 2. Methods

A systematic search was performed in PubMed regarding cardiac electroporation using the search words ‘electroporation AND atrial OR electroporation AND ventricular OR irreversible electroporation OR IRE OR pulsed field ablation’ for studies published in English.

## 3. Mechanisms of PFA

**Figure 1:**
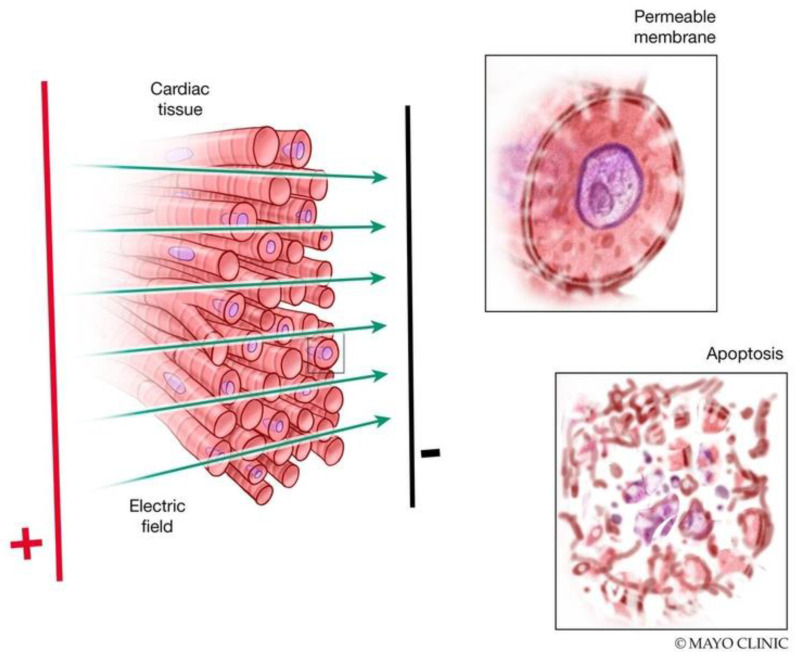
Mechanisms of electroporation. Delivery of a strong pulsed electric field (PEF) results in pore formation and increased cell membrane permeability. These changes may be reversible with a return to normal cell function or irreversible with progression to cell death.

**Figure 2:**
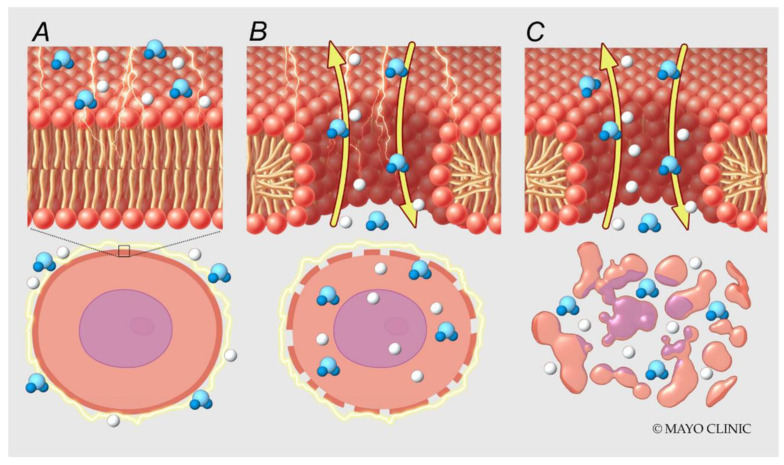
Molecular mechanisms of electroporation. High voltage is applied across electrodes (**A**). Water molecules move along local electric field gradients resulting in pore formation (**B**) and cell death (**C**).

**Figure 3:**
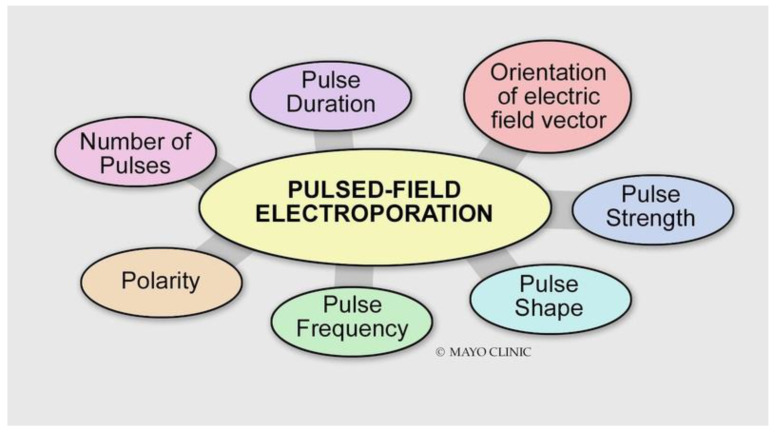
Various energy delivery parameters can affect the magnitude of the electroporation effect.

Electroporation is achieved by applying high voltages or currents across electrodes. In 1982, Scheinman et al. first described the use of high-energy direct current (DC) shock for ablation of the atrioventricular node in humans [1]. The main difference between electroporation and procedures in the 1980s is the lower current density at the electrode surface, decreasing the risks of arcing and barotrauma. Electroporation can be reversible or irreversible. The induced electric field results in the accumulation and redistribution of charges across the cell membrane, the formation of pores in the cell membrane, and the loss of cellular homeostasis. If the cell cannot return to its normal function, it then dies via various programmed cell death pathways (Figure 1). The mechanism of pore formation during electroporation was studied using molecular dynamic simulations and explained by local electric field gradients at the water–lipid interface [2]. These effects result in water defects penetrating the bilayer interior, which further increases the local electric field [2] (Figure 2, Video S1). On a cellular level, the exact mechanism of cell death is not fully understood, but it has been attributed to adenosine triphosphate (ATP) depletion, proteolysis, and calcium overload. The apoptotic effect of electroporation depends on several factors, including the energy delivery parameters, the electrode–tissue interface, and the tissue characteristics (Figure 3). It is worth noting that even though cardiac electroporation is often described as a non-thermal ablation modality, there is still some tissue heating with the high-voltage pulses delivered, which is proportional to the product of the local electric field and current density [3].

### Energy Delivery Parameters

1. **Voltage:** Different types of tissue have different electroporation thresholds [4]. Atrial cardiomyocytes have lower electroporation thresholds than surrounding structures (400 V/cm) [5]. Increasing the voltage increases the electroporation effect.
2. **Pulse duration:** Increasing the pulse duration increases the magnitude of the electric field delivered, which results in a larger electroporation effect.
3. **Number of pulses (frequency):** The efficacy of biphasic PFA is dependent on the frequency of the waveform. Higher frequencies result in smaller PFA lesions [6]. This may be explained by the decreased ability of the applied electric field to generate a sufficiently high transmembrane potential for electroporation to occur.
4. **Biphasic versus monophasic:** Monophasic energy delivery results in a larger electroporation effect as compared with biphasic energy delivery at the expense of substantial skeletal muscle, diaphragmatic engagement, and pain [6,7,8].
5. **Bipolar versus unipolar:** A unipolar configuration creates deeper lesions at the expense of significant skeletal muscle contraction and pain.

Other factors that can affect lesion size:

1. **Contact:** Effective PFA is dependent on proximity, but not necessarily greater electrode contact force to the target tissue. Hence, PFA may be more successful at ablating trabeculated regions and sites without optimal contact and stability. Even though PFA is not contact-dependent per se, several studies have shown that at constant pulsed electric field current and pulse duration, lesion depth increased significantly with increasing contact force [9,10].
2. **Electrode surface area:** The smaller the surface area of the catheter electrode is, the greater the electroporation effect.
3. **Electrode arrangement:** Lesion depth is greater with an ablation hoop than with a single ablation electrode. The large total electrode surface area of a multielectrode hoop catheter decreases the risk of arcing. The current delivered by a multielectrode hoop is predominantly directed outward rather than to the center of the hoop [11].
4. **Cellular orientation in relation to the direction of the electric field:** Using nanosecond pulses, there is a greater electroporation effect with a perpendicular orientation than parallel. With millisecond pulses, there is a greater electroporation with a parallel orientation than perpendicular. Using microsecond pulses, cells of both orientations were electroporated to the same extent [12].

Although considerable progress has been made with moving PFA from bench to bedside for the management of atrial arrhythmias, there are limited data on PFA for ventricular ablation. Most devices are currently geared towards thin-walled atria, and thicker ventricular tissue may require different PFA configurations. For instance, bipolar ablation in the atria may not be as contact-dependent but ventricular tissue may benefit from unipolar ablation which is more contact-dependent.

## 4. Efficacy of PFA

### 4.1. PFA for Atrial Arrhythmias

Table 1 and Table 2 summarize the main findings of pre-clinical and clinical studies assessing the outcomes of cardiac electroporation for atrial ablation and the management of atrial arrhythmias. In 2007, Lavee et al. first described the intentional use of irreversible electroporation (IRE) as an ablation energy source for surgical epicardial atrial ablation [13]. Since then, several studies using different catheters, pulsed electric field (PEF) generators, and setups have shown the efficacy of IRE in creating transmural atrial lesions while sparing adjacent structures.

In 2018, Reddy et al. reported the first clinical experience with PFA for the management of paroxysmal atrial fibrillation (AF) [19]. This study showed that PEF-based ablation of the pulmonary veins (PVs) and left atrium (LA) was safe and feasible, whether it was performed endocardially or epicardially. In 2019, the results of the IMPULSE (A Safety and Feasibility Study of the IOWA Approach Endocardial Ablation System to Treat Atrial Fibrillation) and PEFCAT (A Safety and Feasibility Study of the FARAPULSE (Boston Scientific) Endocardial Ablation System to Treat Paroxysmal Atrial Fibrillation) trials were published [20]. The ablation protocol underwent consecutive modifications: from monophasic to biphasic pulses, followed by optimization of the biphasic waveform morphology and pulse sequence composition with improvement in durable PVI success rates at 3-month follow-up. The 12-month freedom from arrhythmia was 87.4 ± 5.6% [20]. These findings were in line with the 1-year outcomes of the IMPULSE, PEFCAT, and PEFCAT II (Expanded Safety and Feasibility Study of the FARAPULSE Endocardial Multi Ablation System to Treat Paroxysmal Atrial Fibrillation) studies [24].

In 2020, Reddy et al. demonstrated that PFA was safe and feasible for the management of persistent AF with durable lesions on remapping at two and a half-month follow-up [21]. The PVs and LA posterior wall remained isolated in 96 and 100% of the cases, respectively [21]. Isolation was defined by entrance block. Similarly, Schiavone et al. showed that LA posterior wall isolation (PWI) using PFA is safe and feasible in patients with persistent AF [33]. During a median follow-up of 273 days, 41 (16.5%) patients had an arrhythmic recurrence with no differences noted among the ablation strategies (PVI only versus PVI + LAPWI) [33]. Mitral isthmus ablation has also been reported to be feasible with the FARAPULSE PFA system (Boston Scientific) with a 4.4% risk of coronary artery spasm [30]. However, this catheter appears to be less suited for ablation of the mitral isthmus and the anterior line as compared with the LA posterior wall and roof [28].

In 2022, the PUSLED AF Pilot trial was published showing the safety and feasibility of acute PVI with the PulseSelect PFA system (Medtronic) in patients with paroxysmal as well as persistent AF [27]. This was followed by the PULSED AF Pivotal trial in 2023, which showed a freedom from arrhythmia recurrence of 66.2% in patients with paroxysmal AF and 55.1% in patients with persistent AF at 1 year [29]. The rate of primary safety adverse events was low at 0.7% [29].

In a recent meta-analysis by Aldaas et al., which included six comparative studies and a total of 1012 patients comparing PFA to other thermal energy sources, PFA was associated with shorter procedural times and longer fluoroscopy times [36]. There was no difference in periprocedural complications or rates of recurrent AF between the two groups [36]. This was consistent with the results of the ADVENT trial, which is a randomized controlled trial that compared PFA to conventional thermal ablation [32]. The trial showed that in patients with paroxysmal AF undergoing catheter ablation, PFA was non-inferior to conventional thermal ablation with respect to freedom from a composite of initial procedural failure, documented atrial tachyarrhythmia after a 3-month blanking period, antiarrhythmic drug use, cardioversion, or repeat ablation and with respect to periprocedural serious adverse events at 1 year [32]. When compared to cryoballoon PVI in particular, PFA had similar acute and chronic success rates but was associated with a shorter procedure time and no phrenic nerve palsies [31]. The longer fluoroscopy time with PFA is expected to improve with the integration of PFA systems with three-dimensional mapping systems [34].

### 4.2. PFA for Ventricular Arrhythmias

Table 3 and Table 4 summarize the main findings of pre-clinical and clinical studies assessing the outcomes of cardiac electroporation for ventricular ablation and management of ventricular arrhythmias. In experimental animal studies, epicardial electroporation has been shown to be feasible and effective at creating transmural ventricular lesions with adequate contact, which is not surprising in the absence of a blood pool that causes significant current leakage [37,38,39]. However, it remains unclear whether electroporation can create significant myocardial lesions through thick areas of epicardial fat. Because the mechanism of lesion formation in electroporation is not primarily dependent on thermal injury, there is no sparing of myocardial injury surrounding arteries. The ability of epicardial PFA to produce transmural lesions while preserving the coronary arteries is highly appealing [37,38,39].

Regarding endocardial ablation, several animal studies have shown that PFA is effective at creating transmural lesions in healthy ventricular myocardia as well as scarred ventricular myocardia, whether the scar was due to a prior infarction or ablation [45,46]. In scars, lesion depth with PFA was greater than radiofrequency ablation (RFA) [46]. PFA of common locations of ventricular arrhythmias including the interventricular septum, papillary muscles, and left ventricular summit via the distal coronary sinus has also been shown to be feasible and effective at creating deep lesions [47,56,57]. In the left ventricular summit and papillary muscles, PFA resulted in larger lesions and fewer steam pops as compared with RFA [57].

## 5. Safety of PFA

**Figure 4:**
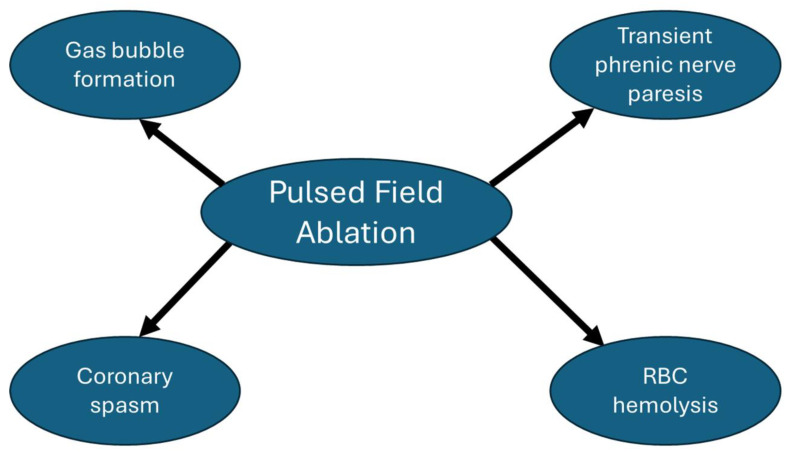
Pulsed field ablation energy-specific adverse effects.

Figure 4 summarizes the main energy-specific adverse effects that have been reported with PFA. Based on the MANIFEST-17K study, which included 17,642 patients with AF undergoing PFA, PFA energy-specific adverse events included transient phrenic nerve paresis (0.06%), coronary spasm (0.14%), and hemolysis-related renal failure (0.03%) [62].

### 5.1. Gas Bubble Formation

Gas bubble formation is commonly reported with PFA and seen on intraprocedural intracardiac echocardiography imaging. Potential explanations for gas bubble formation with PFA include heating, electrolysis, and the de-gasification or displacement of gases from the blood. In patients treated with PFA for the management of AF, the incidence of asymptomatic thromboembolic cerebral events or lesions detected on brain imaging ranged between 3 and 9% [63,64]. The amount of gas bubble formation depends on several parameters of energy delivery. It is directly related to the delivered charge. Anodal IRE applications result in less gas formation than cathodal IRE applications and radiofrequency applications [65]. Sub-RFA alternating currents (AC) pulses have also been shown to avoid electrolysis-induced gas bubble formation [66].

### 5.2. Phrenic Nerve Injury

Reported findings in the literature regarding the effect of electroporation on nerves are variable with some describing a minimal effect [67] and others describing transient damage with recovery at 7 weeks [68]. The preservation of the endoneurium architecture and proliferation of Schwann cells seen histologically post-ablation reflect the potential for axonal regeneration [69,70]. Van Driel et al. demonstrated that energy levels that could create myocardial lesions spared the phrenic nerve without histologic or functional evidence of phrenic nerve damage [71]. The proximity of the catheter to the phrenic nerve and the PFA dose level are predictors of phrenic nerve response to PFA [72]. Recently, Ollitrault et al. reported transient phrenic nerve stunning without phrenic nerve palsy at the end of the procedure and at hospital discharge in 64% of patients undergoing isolation of the superior vena cava using a pentaspline PFA catheter [73].

### 5.3. Esophageal Injury

Neven et al. demonstrated that the esophageal architecture was unaffected 2 months after delivering IRE directly to the esophageal adventitia [74]. Similarly, Song et al. showed no histopathologic changes to the esophagus at 4 weeks and 16 weeks after monophasic, bipolar IRE of the esophagus [75,76]. In an in vivo porcine esophageal injury model, ablation at areas of esophageal contact resulted in no histopathologic esophageal changes with PFA and a spectrum of esophageal lesions with RFA [77]. In patients with AF undergoing PFA, a dose-dependent rise in esophageal temperature has been reported [78]. The long-term implications of this finding need further evaluation.

### 5.4. Coronary Artery Damage

Animal studies showed that epicardial IRE did not result in coronary vessel luminal narrowing at 3 weeks and 3 months post-ablation [37,39]. Conversely, intracoronary PFA can lead to fixed coronary stenosis [79]. Furthermore, there have been several reports of coronary artery spasm noted transiently post-PFA [38,40,79]. The exact mechanism of coronary artery spasm post-PFA is not entirely known. Potential explanations include transient activation of vascular smooth muscles by the delivered PEF [80]. This phenomenon is responsive to nitroglycerin administration either pre- or post-ablation [81,82].

### 5.5. Pulmonary Vein Stenosis

In animal studies, multiple circumferential electroporation applications inside the ostia of the pulmonary veins (PVs) did not result in PV narrowing or stenosis at 1-month and 3-month follow-up whereas RFA did [83,84]. In patients with AF undergoing catheter ablation, Kuroki et al. demonstrated that the incidence and severity of pulmonary vein (PV) narrowing and stenosis were significantly lower with PFA as compared to RFA [85]. This may be explained by mechanistic differences in the ablation and healing processes between PFA and RFA.

### 5.6. Hemolysis

Lysis of red blood cells is common with PFA and has been shown to occur in a dose-dependent manner [86]. In a study comparing the risk of hemolysis during PVI with PFA versus RFA, significant renal injury was uncommon with a number of 70 PFA lesions [87]. The PFA system that was used in this study was the Farapulse PFA system (Boston Scientific).

## 6. PFA versus Conventional Ablation Modalities

Table 5 summarizes the technical aspects of the two PFA systems that are currently available on the market in the US. PFA has several advantages and disadvantages when compared to thermal ablation modalities such as RFA and cryoablation. It has been shown to carry a lower risk for collateral damage, reducing complications such as esophageal injury and pulmonary vein stenosis. Furthermore, it can induce immediate cell death, and this rapid response may shorten procedure times compared to RFA and cryoablation, which require time to reach effective thermal thresholds.

As PFA technology is still evolving, there is less standardization in devices and protocols compared to the well-established RFA and cryoablation systems. Unlike thermal modalities that often allow for real-time temperature and electrogram monitoring, PFA lacks direct indicators for successful ablation during the procedure, making it more difficult to assess the completeness of lesion formation in real-time. Furthermore, while PFA carries a lower risk for collateral damage, there is still a risk of unintended effects, such as coronary spasms and gas bubble formation, which require further research and the refinement of ablation parameters to mitigate. Lastly, the PFA systems available currently use larger sheaths which carry a higher risk for vascular access issues, cardiac perforation, and air emboli.

## 7. Future Directions

Despite the high level of enthusiasm, there are still many aspects of cardiac electroporation that need to be understood and optimized. The notions of the non-thermal nature and tissue selectivity of cardiac electroporation have been debunked. Future research should aim to enhance our ability to titrate the ablative effects of cardiac electroporation and distinguish between acute outcomes of reversible versus IRE. Additionally, efforts should focus on mitigating potential energy-specific adverse effects, including gas bubble formation and coronary spasm, optimizing PFA configurations for ventricular ablation, and integration of PFA systems with mapping systems.

## 8. Conclusions

In conclusion, cardiac electroporation represents a promising new modality in the field of cardiac electrophysiology, offering potential safety advantages over traditional ablation techniques. By leveraging the mechanisms of reversible and IRE, this technology provides a novel approach to achieve precise and targeted tissue ablation with minimal collateral damage. Early studies and clinical trials assessing the outcomes of PFA for the management of arrhythmias have demonstrated encouraging results. However, further research is needed to optimize PFA protocols, understand long-term effects, and establish comprehensive guidelines for clinical application. As the field continues to evolve, cardiac electroporation may become a valuable addition to the therapeutic arsenal for treating cardiac arrhythmias.

## Figures and Tables

**Table 1:** Summary of pre-clinical studies on atrial PFA.

Study Authors	Year	Sample	Catheter	PFA Settings	Ablation Location	Results
**Lavee et al. [13]**	2007	5 pigs	Two 4-cm long parallel electrodes.	A sequence of 8, 16, or 32 DC pulses of 1500 to 2000 V, 100 µs each, at a frequency of 5 per sec.	Epicardial	Atrial lesions were transmural. Mean lesion depth was 0.9 cm.
**Stewart et al. [14]**	2018	6 pigs	A nine-electrode circular array PV ablation catheter (PVAC GOLD; Medtronic).	Biphasic pulse trains with a pulse width of 100 µs for each phase and 200 µs interpulse pauses. Five pulse trains of 60 pulses per train at 500 V delivered over 10 s.	Endocardial	Intracardiac PFA was feasible with acute electrical effects (reduction of electrogram amplitudes and loss of bipolar capture). At 2 weeks, PFA resulted in transmural and homogenous fibrosis.
**Ye et al. [15]**	2021	Isolated rat myocardial and smooth muscle cells of 3 pigs	An 8 Fr PFA catheter.	1600 V/cm in bipolar short pulse mode (the forward pulse width was 5 µs; reverse pulse width was 3 µs; pulse interval was 3 µs) with 1000 pulses and 8-A current.	PV Renal artery branches for safety data	Bidirectional PFA was effective at isolating the PVs with maintenance of cell–cell connection and a reduction of muscle contraction.
**Stewart et al. [16]**	2021	6 pigs	A nine-electrode circular array PV ablation catheter (PVAC GOLD; Medtronic).	**Low PFA dose:** trains of biphasic pulses, each of these having +700 V pulse followed by a reverse polarity −700 V pulse. **High PFA dose:** trains of biphasic pulses, each of these having +1500 V pulse followed by a reverse polarity −1500 V pulse.	SVC, RAA, RSPV	PFA resulted in complete circumferential replacement fibrosis at 4-weeks post-ablation with no extracardiac damage.
**Hsu et al. [17]**	2022	8 pigs	A 7.5 Fr PFA circular catheter (Biosense Webster).	Biphasic, bipolar PFA. Total application duration: 250 ms.	Left and right atria One PV per animal was isolated while a second PV was ablated deep inside the vein, targeting intensive narrowing.	PV narrowing was not observed acutely or at follow-up, even when the ablation was performed deep inside the vein. There was no injury to adjacent structures. All veins remained isolated upon remapping at 1 month.
**Koruth et al. [18]**	2023	29 canines 3 swine	A multielectrode spherical array with 16 flat ribs and 122 gold-plated electrodes.	A train of μs pulses. Voltage: 1600–2000 V. PFA duration: 3–4 s.	PV ablation in canines. SVC and esophageal ablation in swine.	Circumferential, linear, and focal mapping and ablation were achieved with this novel catheter using PFA and RFA.

**Abbreviations:** DC: direct current, Fr: French, μs: microsecond, ms: millisecond, ns: nanosecond, PFA: pulsed field ablation, PV: pulmonary vein, RAA: right atrial appendage, RSPV: right superior pulmonary vein, sec: second, SVC: superior vena cava, V: Volt.

**Table 2:** Summary of clinical studies on atrial PFA.

Study Authors	Year	Sample	PFA Catheter	PFA Settings	Ablation Location	Results
**Reddy et al. [19]**	2018	22 patients with paroxysmal AF.	A custom 12 Fr over-the-wire PEF endocardial ablation catheter A linear epicardial PEF ablation catheter.	Bipolar PFA A train of ms pulses delivered over a few sec. Voltage range: 900 to 2500 V.	Endocardial: PVI, Epicardial: PVs and posterior left atrium.	Endocardial PV isolation was successful in 15/15 (100%) patients. Surgical box lesions were successful in 6/7 (86%) patients. There were no complications.
**Reddy et al. [20]** **The IMPULSE and PEFCAT studies**	2019	81 patients with paroxysmal AF.	A 12 Fr multielectrode PFA catheter (Farawave, Boston Scientific).	Monophasic PFA: 900 to 1000 V. Biphasic PFA: 1800 to 2000 V.	PVI in all patients.	With successive waveform modifications, durability at 3 months improved from 18% to 100% of patients with all PVs isolated. The 12-month estimate of freedom from arrhythmia was 87.4 ± 5.6%. There was 1 procedure-related tamponade.
**Reddy et al. [21]**	2020	25 patients with persistent AF.	A 12 Fr multielectrode PFA catheter (Farawave, Boston Scientific).	Bipolar PFA A train of μs pulses.	PVI in all patients PWI in 24 (96%) patients, CTI in 13 (52%) patients	Two and a half months later, all patients had remapping. PVI, PWI, and CTI lesions were isolated in 96, 100, and 100% of the cases, respectively.
**Reddy et al. [22]**	2020	76 patients with AF, 55 (72.4%) patients with paroxysmal AF, 21 (27.6%) patients with persistent AF.	A 7.5 Fr lattice catheter.	Biphasic PFA A train of μs pulses delivered over 3 to 5 s, with total current delivery 24 and 32 A.	PVI in all patients **Linear ablations** - Mitral: 14 (18.4%) patients, - Left atrium roof: 34 (44.7%) patients, - Cavotricuspid isthmus: 44 (57.9%) patients.	A novel lattice-tip catheter allowed for safe and effective ablation of AF with a combined RFA/PFA approach or an entirely PFA approach.
**Loh et al. [23]**	2020	10 patients with AF, 6 (60%) patients with paroxysmal AF, 4 (40%) patients with persistent AF, 1 (10%) patient with longstanding persistent AF.	A custom non deflectable 14-polar circular IRE ablation catheter with a variable hoop diameter.	6 ms, 200 J DC IRE applications. A minimum of 2 IRE applications per PV.	PVI in all patients.	All PVs were successfully isolated with a mean of 2.4 ± 0.4 IRE applications per PV. Acute bidirectional PVI can be achieved by single pulse IRE ablation.
**Reddy et al. [24]**	2021	121 patients with paroxysmal AF.	A 12 Fr multielectrode PFA catheter (Farawave, Boston Scientific).	Monophasic PFA: 900 to 1000 V. Biphasic PFA: 1800 to 2000 V. Number of pulses: 4–10 per application.	PVI in all patients +/− CTI ablation if clinically indicated.	PV remapping, performed in 110 patients at 2–3 months, showed durable PVI in 84.8% of PVs (64.5% of patients) and 96.0% of PVs (84.1% of patients) treated with the optimized biphasic energy PFA waveform. The 1-year for freedom from any atrial arrhythmia for the entire cohort and for the optimized biphasic energy PFA waveform cohort were 78.5 ± 3.8% and 84.5 ± 5.4%, respectively.
**Kawamura et al. [25]**	2021	59 patients with AF, 20 patients (33.9%) with PFA, 39 (66.1%) patients with thermal ablation.	A 12 Fr multielectrode PFA catheter (Farawave, Boston Scientific).	Bipolar PFA A train of μs pulses.	PVI in all patients.	The areas of PV antral isolation were not significantly different between the PFA and thermal ablation groups. After propensity matching, PFA resulted in a larger isolation area at the LIPV than RFA.
**Kawamura et al. [26]**	2021	20 patients with paroxysmal AF.	A 12 Fr multielectrode PFA catheter (Farawave, Boston Scientific).	Biphasic PFA (PEFCAT protocol).	PVI in all patients.	The level of PV antral isolation after PFA persisted at a median of 84 days.
**Verma et al. [27]** **The Pulsed AF Pilot trial**	2022	38 patients with AF, 35 (92.1%) patients with paroxysmal AF, 3 (7.9%) patients with persistent AF.	A circular multielectrode array catheter (PulseSelect, Medtronic).	Bipolar, biphasic PFA.	PVI in all patients +/− CTI ablation if clinically indicated.	There was 100% PVI with no PFA-system related adverse events.
**Kueffer et al. [28]**	2022	22 patients with documented LA reentry.		Bipolar, biphasic PFA. A train of μs pulses. Peak voltage: 2000 V.	Roof line and posterior wall in 20 (90.9%) patients. Anterior line in 13 (59.1%) patients. Mitral isthmus line in 6 (27.3%) patients.	Additional RFA was necessary for 2 anterior lines (15%) and 3 mitral isthmus lines (50%). Bidirectional block was present across all roof lines, 92% of anterior lines, and 83% of mitral isthmus lines.
**Verma et al. [29]** **The Pulsed AF Pivotal trial**	2023	300 patients with AF. 150 (50%) patients with paroxysmal AF. 150 (50%) patients with persistent AF	A circular multielectrode array catheter (PulseSelect, Medtronic).	One application was defined as 4 biphasic, bipolar pulse trains, each lasting 100 to 200 ms at 1400 to 1500 V from baseline to peak.	PVI in all patients.	PFA was effective at 1 year in 66.2% of patients with paroxysmal AF and 55.1% of patients with persistent AF. The rate of primary safety adverse events was low at 0.7%.
**Davong et al. [30]**	2023	45 patients with persistent AF.	A 12 Fr multielectrode PFA catheter (Farawave, Boston Scientific).	Bipolar, biphasic PFA.	PVI + LAPWI + MI ablation in all patients.	Complete MI block was achieved in all patients. Complications occurred in 3 (6.6%) patients including coronary artery spasm (2 patients) and air embolism (1 patient).
**Urbanek et al. [31]**	2023	400 patients with AF. 243 (60.7%) patients with paroxysmal AF.	A 12 Fr multielectrode PFA catheter (Farawave, Boston Scientific).	Bipolar, biphasic PFA. A train of 5 consecutive waves in 2.5 s of total ablation. Voltage: 2000 V.	PFA PVI in 200 (50%) patients. Cryoballoon PVI in 200 (50%) patients.	PFA had similar efficacy, compared to cryoballoon ablation, but led to shorter procedure times and no phrenic nerve palsies. 12-month clinical success rates were similar between the 2 groups.
**Reddy et al. [32]** **The ADVENT trial**	2023	607 patients with paroxysmal AF.	A 12 Fr multielectrode PFA catheter (Farawave, Boston Scientific).	Bipolar, biphasic PFA. A total of 8 PFA applications per PV. Each PFA application consists of 5 packets of pulses delivered over 2.5 s. Voltage: 1800, 1900 or 2000 V.	PVI in 305 patients with PFA and 302 patients with thermal ablation, CTI ablation in 70 (23%) patients with PFA, and 86 (28.5%) patients with thermal ablation. Additional ablation outside the PVs in 5 (1.6%) patients with PFA and 16 (5.3%) patients with thermal ablation.	PFA was noninferior to thermal ablation with respect to freedom from a composite of initial procedural failure, documented atrial tachyarrhythmia after a 3-month blanking period, antiarrhythmic drug use, cardioversion, or repeat ablation (73.3% with PFA versus 71.3% with thermal ablation) and with respect to periprocedural serious adverse events (2.1% with PFA versus 1.5% with thermal ablation) at 1 year.
**Schiavone et al. [33]**	2024	249 patients with AF. 54 (21.7%) patients with longstanding persistent AF.	A 12 Fr multielectrode PFA catheter (Farawave, Boston Scientific).	Bipolar, biphasic PFA.	PVI only in 106 (42.6%) patients. PVI + LAPWI in 142 (57%) patients. Additional lesions only in 1 (0.4%) patient.	LA PWI with PFA was safe and feasible. During a median follow-up of 273 days, 41 (16.5%) patients had an arrhythmic recurrence. There was no difference among the ablation strategies (PVI-only versus PVI + LAPWI versus redo patients).
**Duytschaever et al. [34]**	2023	226 patients with paroxysmal AF.	A variable loop circular catheter (Biosense Webster).	Bipolar, biphasic PFA. Voltage: 1800 V A train of μs pulses for a total application duration of 250 ms.	PVI in all patients.	The novel mapping integrated PFA system was safe and effective.
**Turagam et al. [35]**	2023	1568 patients with AF, 1019 (65%) patients with paroxysmal AF, 502 (32%) patients with persistent AF.	A 12 Fr multielectrode PFA catheter (Farawave, Boston Scientific).	Bipolar, biphasic PFA. A train of μs pulses. Each PFA application consists of 5 packets of pulses delivered over 2.5 s.	PVI in all patients. Additional ablation: Posterior wall: 173 (11%). MI: 37 (2.4%). CTI: 84 (5.4%). Roof line: 21 (1.3%).	The 1-year freedom from atrial arrhythmias was 78.1% (81.6% in paroxysmal AF versus 71.5% in persistent AF, *p* = 0.001). There were acute major adverse events in 1.9% of patients.

**Abbreviations:** AF: atrial fibrillation, CTI: cavotricuspid isthmus, Fr: French, IRE: irreversible electroporation, LA: left atrium, LIPV: left inferior pulmonary vein, MI: mitral isthmus, μs: microsecond, ms: millisecond, ns: nanosecond, PFA: pulsed field ablation, PV: pulmonary vein, PVI: pulmonary vein isolation, PWI: posterior wall isolation, RFA: radiofrequency ablation, sec: second, V: Volt.

**Table 3:** Summary of pre-clinical studies on ventricular PFA.

Study Authors	Year	Sample	Catheter	PFA Settings	Ablation Location	Results
**Neven et al. [38]**	2014	5 pigs.	Custom circular octapolar ablation catheter, 12 mm diameter, 7 Fr, 2 mm electrodes, surface area 115 mm^2^.	Single cathodal 50, 100, or 200 J, monophasic.	Epicardial.	Lesion size significantly correlated to magnitude of the electroporation application. With 200 J, mean width was 19.8 mm and mean depth was 11.9 mm. Post-ablation coronary angiography showed coronary spasm.
**Neven et al. [40]**	2014	5 pigs.	Custom linear suction device with a single 35 × 6 mm electrode inside a 42 mm long and 7 mm wide plastic suction cup.	Single cathodal applications of 30, 100, or 300 J (6 ms).	Epicardial.	Magnitude of the electroporation application significantly contributed to lesion size. With 300 J, mean width was 17.1 and mean depth was 8 mm.
**Livia et al. [41]**	2018	8 ex vivo Langendorff models of canine hearts.	Modified 8-mm RF ablation catheter.	Unipolar applications at escalating voltages, frequency of 1 Hz, pulse duration of 90 μs.	Endocardial (Purkinje tissue).	Purkinje tissue can be ablated with irreversible electroporation without myocardial damage.
**Azarov et al. [42]**	2019	Isolated murine ventricular cardiomyocytes.		200 ns trapezoidal pulses.		nsPEF caused calcium entry into cardiac myocytes (including routes other than voltage-gated calcium channels) and slow sustained depolarization.
**Koruth et al. [43]**	2020	4 pigs.	12 Fr deflectable PFA catheter.	Single applications with a voltage of 2200 V (biphasic) and spanning 6 heart beats.	Endocardial. Left and right ventricles.	Mean lesion width was 22.6 mm and mean lesion depth was 6.5 mm.
**Neven et al. [44]**	2021	7 pigs.	Custom linear suction device with a single 35 × 6 mm electrode inside a 42 mm long and 7 mm wide plastic suction cup.	Single cathodal 200 J applications.	Epicardial. Right ventricle (base to apex).	In the first hour, there was contraction band necrosis and edema. After 3 weeks, there was sharply demarcated loose connective tissue. After 3 months, there was more fibrotic scar tissue. Arteries and nerves were unaffected.
**Nakagawa et al. [9]**	2022	2 pigs	7 Fr catheter with a 3.5 mm electrode and contact force sensor (Tacticath, Abbott)	Unipolar delivery RV: 28 A for 1.4 ms LV: 35 A for 1.6 ms	Endocardial. 3 contact settings: Low, 4–12 g, Moderate, 16–30 g, High, 35–55 g.	At a constant pulsed electric field current and pulse duration, greater contact force increased lesion depth significantly.
**Kawamura et al. [45]**	2022	Pigs.	Focal lattice-tip catheter.	Proprietary biphasic monopolar PFA applications.	Endocardial and epicardial. Scar created prior PFA (4 weeks earlier).	Repetitive PFA applications improved lesion depth. Epicardial PFA is effective and generates lesions with comparable dimensions. Endocardial scarring up to 4 mm depth did not impact subsequent PFA from penetrating the myocardium beyond the scar.
**Im et al. [46]**	2022	10 pigs.	Linear quadripolar (FOCAL) and multispline 8-pole catheter (BASKET).	Bipolar, biphasic PFA was delivered for 2.5 s x4 applications/site.	Endocardial. Left ventricular healthy and scarred myocardium.	There was no difference in lesion depth between the focal and multispline catheters. Lesion width was greater with the multispline catheter. In scar, lesion depth for PFA was greater than RFA.
**Van Zyl et al. [47]**	2022	8 canines.	Commercial ablation catheters (EPT Blazer II, Boston Scientific) for epicardial ablation.	ns PFA and μs deliveries. ns PFA settings: proprietary data, μs PFA: monophasic, 1000–1500 V, 100 μs, 40–60 pulses.	Endocardial. Interventricular septum, right and left ventricles.	Bipolar PFA of the interventricular septum is feasible and can produce near transmural lesions. Myocardial stunning and conduction system injury occurred transiently.
**Tan et al. [48]**	2022	5 canines.	Active fixation pacing leads.	ns PFA and μs deliveries. With 1 lead, energy delivery ranged from 0.64 to 7.28 J. With 2 leads, energy delivery ranged from 56.3 to 144.9 J.	Endocardial Right ventricular septum.	Ventricular PFA is safe and feasible using active fixation leads. Reversible changes were seen with lower energies whereas durable lesions were seen with higher energies.
**Verma et al. [49]**	2022	4 pigs.	7 Fr catheter with a 3.5 mm electrode and contact force sensor (Tacticath, Abbott).	Monopolar, biphasic PFA 19-, 22-, and 25- A settings.	Endocardial. Right and left ventricles.	Focal monopolar PFA catheters are able to generate deep lesions. Mean lesion depths were 5.7 mm, 7.2 mm, and 8.2 mm for the 19-, 22-, and 25- Amp settings, respectively.
**Chaigne et al. [50]**	2022	Isolated rat left ventricular myocytes.	2 platinum electrodes (4 mm spacing).	Monophasic deliveries at different voltages and pulse durations.	Isolated left ventricular myocytes.	Electroporation resulted in an immediate increase in intracellular calcium, which was dependent on the voltage delivered. Lethal EP voltage threshold was lower in myocytes oriented perpendicular than parallel to the electric field using 100 μs pulses while an opposite effect was found using 10 msec pulses.
**Kawamura et al. [51]**	2023	6 pigs with scar (5 with coronary artery occlusion and 1 with RF). 2 controls.	8 Fr focal PFA catheter.	Bipolar, biphasic PFA. Voltage: 2000 V.	Endocardial. Epicardial in the 2 healthy pigs.	No significant difference between PFA lesion depth at the infarct border (mean of 5.9 mm) and healthy myocardium (mean of 5.7 mm). PFA penetration of both infarct and iatrogenic RFA scar was observed. Dark-blood LGE (shorter T1 times) allowed for improved endocardial order detection as well as lesion boundaries.
**Kawamura et al. [52]**	2023	Healthy swine.	Lattice tip ablation catheter (Sphere-9, Affera). 7.5 Fr bidirectionally deflectable shaft with a lattice tip containing 9 mini-electrodes.	Monopolar biphasic waveform (2000 V, 4-s duration deliveries).	Endocardial PFA and RFA	At 1-h post-PFA, myocytes were disrupted. Swollen mitochondria with degenerating nuclei and condensed chromatic were seen. At 4 h post-PFA, there was worsening edema. Histologic features of PFA included sparing of vessels and sharp lesion margins.
**Kos et al. [53]**	2023	Ex vivo perfused porcine and human left ventricles.	Pair of parallel needle electrodes.	2 pulse waveforms: Proprietary biphasic waveform (Medtronic) and monophasic 48 × 100 μs pulses.	Ex vivo perfused porcine and human left ventricles.	Median threshold was 535 V/cm in porcine and 416 V/cm in human donor hearts. The median threshold was 368 V/cm in porcine hearts for 48 × 100 μs pulses. Treatments in humans with parameters optimized in pigs should result in equal or greater lesions.
**Aryana et al. [54]**	2023	10 healthy pigs.	6-/8- Fr linear and spiral PFA/mapping catheters (CRC EP).	Bipolar PFA (>2000 V), biphasic, μs pulses.	Endocardial and epicardial. Right and left ventricles.	This novel PFA system using linear and spiral PFA catheters was able to create large and durable lesions without significant microbubbling, ventricular arrhythmias, or thromboembolism.
**Di Biase et al. [10]**	2024	11 swine (derivation). 6 swine (validation).	CF-sensing OMNYPULSE catheter (Biosense Webster).		Endocardial. Left and right ventricles.	Mean lesion depth was 3.5 mm ad width was 12.0 mm. More than contact force and PFA dose alone, it was their combined effect that impacted lesion depth (PFA index).
**Younis et al. [55]**	2024	3 pigs with long duration RFA after RFA. 3 pigs with PFA after RFA.	8 Fr Farapoint focal catheter (Boston Scientific).	4 PFA applications for each lesion, 2000 V. For RFA, targeting a min impedance drop of 40 ohms over 60 s and stopped when a max of 50 ohms was reached.	Endocardial. Left ventricle, PFA following RFA.	PFA created lesions that are deeper than RFA when ablating over superficial RFA lesions.
**Nies et al. [56]**	2024	13 swine.	A lattice-tip catheter (Sphere-9, Medtronic).	Biphasic, monopolar PFA.	Intracavitary papillary muscles and moderator bands. Epicardial targets Midmyocardial targets in the interventricular septum and LV free wall.	PFA can ablate intracavitary structures and result in deep epicardial and transmural LV lesions.
**Younis et al. [57]**	2024	10 swine.	An investigational dual-energy (RFA/PFA) contact force and local impedance-sensing catheter (modified IntellaNav StablePoint, Boston Scientific).	Monopolar PFA. Voltage: 2000 V 10 packets per application and 4 applications per site.	LV interventricular septum. Papillary muscle LV summit via the distal coronary sinus. LV epicardium.	Compared with RFA, PFA resulted in deeper lesions with fewer steam pops. PFA was associated with higher rates of ST changes with direct epicardial ablation.

**Abbreviations:** A: amperes, Hz: hertz, J: joule, LGE: late gadolinium enhancement, LV: left ventricle, min: minimum, max: maximum, μs: microsecond, ms: millisecond, ns: nanosecond, PFA: pulsed field ablation, RFA: radiofrequency ablation, sec: second, V; Volt.

**Table 4:** Summary of clinical studies on ventricular PFA.

Study Authors	Year	Sample	Catheter	PFA Settings	Ablation Location	Results
**Schmidt et al. [58]**	2021	1 patient.	A 12 Fr multielectrode PFA catheter (Farawave, Boston Scientific).	1800 V, 2.5 s pulse duration.	Endocardial. RVOT.	Successful PVC elimination.
**Adragao et al. [59]**	2023	1 patient.	A 12 Fr multielectrode PFA catheter (Farawave, Boston Scientific).	2000 V for a total of 18 application sites.	Endocardial. Left ventricular basal, mid, and apical septal regions.	Lesion depth was similar in both PFA and RFA sites. Lesion characteristics in the explanted heart were different with apoptotic-like cellular lesions and lack of inflammation in PFA.
**Hansen et al. [60]**	2023	1 patient.	Irrigated-tip contact force sensing catheter approved for PFE delivery.	25 A, R-wave synchronized trains of unipolar pulses.	Endocardial. Superolateral aspect of the mitral valve annulus.	Successful PVC elimination. Since PFA was performed after RFA, there may be potential contribution of pre-treatment with RFA.
**Worck et al. [61]**	2023	1 patient.	A 7.5 Fr SmartTouch catheter (Biosense Webster).	7 QRS-synchronized ablations: The first 2 using 19 A in pulses for 4 s. The final 5 using 22 A in pulses for 7 s.	Endocardial. Posterior RVOT.	Successful and durable PVC elimination.

**Abbreviations:** A: amperes, Fr: French, μs: microsecond, ms: millisecond, ns: nanosecond, PFA: pulsed field ablation, PVC: premature ventricular contraction, RFA: radiofrequency ablation, RVOT: right ventricular outflow tract, sec: second.

**Table 5:** Comparison of the pulsed field ablation systems approved for pulmonary vein isolation.

	PulseSelect PFA System (Medtronic)	Farapulse PFA System (Boston Scientific)
**Ablation size**	25 mm	31 mm or 35 mm
**Electrode number**	9	20
**Ablation electrode configuration**	Fixed (loop)	Variable (flower, basket)
**Waveform**	Biphasic, bipolar	Biphasic, bipolar
**Outer sheath size**	14 Fr	16 Fr
**Inner sheath size**	10 Fr	13 Fr

**Abbreviations:** PFA: pulsed field ablation.

## Supplementary Materials

The following supporting information can be downloaded at: https://www.mdpi.com/article/10.3390/jcm13175191/s1, Video S1: Mechanisms of cardiac electroporation.
